# Myeloperoxidase Content is a Marker of Systemic Inflammation in a Chronic Condition: The Example Given by the Periodontal Disease in Rats

**DOI:** 10.1155/2009/760837

**Published:** 2009-12-31

**Authors:** Celso Martins Queiroz-Junior, Cinthia Mara da Fonseca Pacheco, Allyson Henrique Fonseca, André Klein, Marcelo Vidigal Caliari, Janetti Nogueira de Francischi

**Affiliations:** ^1^Department of Pharmacology, Federal University of Minas Gerais, 31270-100 Belo Horizonte, Brazil; ^2^Department of Health Sciences, Newton Paiva University Center, 30460-720 Belo Horizonte, Brazil; ^3^Department of Pathology, Federal University of Minas Gerais, 31270-100 Belo Horizonte, Brazil

## Abstract

The study aimed to evaluate the suitability of myeloperoxidase (MPO) content as a local indicator of chronic inflammation, using the periodontal disease model. Anesthetized adult male Holtzman rats had their second left maxilar molar tied by a thread for 11 days and were then killed. Blood samples and photographic images from histopathological inflamed and noninflamed (contralateral) neighboring gingivomucosal specimens were collected for cell counts and MPO level analysis. Diseased animals were also treated with pharmacological tools such as the anti-inflammatory drug celecoxib or the opioid morphine. Increased blood neutrophils and local cell numbers characterized diseased animals. However, local MPO content was increased in inflamed and noninflamed tissues from diseased animals. Celecoxib and morphine reduced blood neutrophils and bilateral MPO content, but only celecoxib reduced local cell numbers in diseased animals. It is concluded that MPO content is a good indicator of a systemic rather than a local inflammation in a chronic inflammatory condition.

## 1. Introduction

Migration and accumulation of cells such as polymorphonuclear leukocytes (PMNs) in tissues is considered a characteristic feature of the host response to injuries [1, 2]. Locally, PMNs neutrophils release antimicrobial and inflammatory mediators, which act synergistically to protect and maintain tissues free of pathogens [3, 4]. Myeloperoxidase (MPO), one of such active substances, is a constituent of the azurophilic granules of PMNs that oxidizes chloride ions to the potent bactericidal oxidant hypochlorous acid (HOCl) [5]. MPO secretion by stimulated neutrophils acts, thus, as a host defense mechanism by efficiently mediating microbial killing. Due to its importance during inflammatory processes and for being an indicator of PMN presence in tissues, MPO has been widely used as an inflammatory marker of both acute and chronic conditions [6]. Moreover, MPO changes have also been associated to the severity of many diseases [5, 7, 8]. 

We have previously shown, using an experimental model of periodontal disease [9], a high increase in cell numbers present in gingival tissues as compared with controls, which was taken as an indicator of local inflammation [10–12]. However, characterization of specific cell lines in such inflamed tissues was not accomplished in those studies, given the difficulties to assess individually each cell type. The present study aimed to assess whether PMN neutrophils accounted for the cell influx by means of the local MPO content in inflamed and non-inflamed gingival tissues. Additional animal groups treated with celecoxib and morphine were also evaluated for local MPO content since such drugs have been shown to reduce inflammatory signs detected in this experimental model [11, 12].

## 2. Materials and Methods

### 2.1. The Periodontal Disease Model

In this study we used a standard model of periodontal disease in rats [9], which has been shown to be adjusted to our conditions [11, 12]. In brief, male Holtzman rats (250–300 g) were anaesthetized with a mixture of ketamine-xylazine (116–23 mg/kg, resp.) given by intramuscular (i.m.) route in a volume of 0.1 mL/100 g. A sterile silk ligature was tied around the cervix of the second left maxillary molar tooth, serving as a retention device for subgingival oral microorganisms. This was considered the ipsilateral side. The contralateral side was used as the non-inflamed control. Naive rats were left nonligated. All the animals were killed (using a CO_2_ chamber) on the 11th day of tooth ligation. The handling of the animals was approved by the University Animal Ethics Committee.

### 2.2. Photographic Records

Photographies from control (nonligated) and experimental (ligated) periodontal tissues were obtained at 15-fold magnification through a digital still camera (Sony Cyber-Shot 7.2 megapixels, Sony Electronics Inc., San Diego, CA, USA) adapted to a stereoscopic loupe (ZP101, Prior Scientific Instruments Ltd., Fisher Scientific, Pittsburgh, PA, USA) in order to show macroscopic differences between non-inflamed and inflamed sites.

### 2.3. Determination of Myeloperoxidase (MPO) Activity

Following standard methods [13, 14], the MPO activity was assessed as a quantitative measure to quantify the extent of neutrophil accumulation in whole tissue samples. Gingivomucosal tissue (~20 mm^2^) surrounding the maxillary second molar tooth was removed from ipsi and contralateral sides of the same animals in all groups. After removing, tissue samples were weighed, homogenized in 2 mL cooled (4°C) phosphate buffer (0.1 M NaCl, 0.02 M Na_3_PO_4_, 0.015 M NaEDTA, pH 4.7), and centrifuged at 4°C for 20 minutes at 12.000 rotations per minute (r.p.m.). Pellets were resuspended in 2 mL 0.05 M sodium phosphate buffer (pH 5.4) containing 0.5% hexa-1,6-bisdecyltrimethylammonium bromide (HTAB, Sigma Chemical Co., USA). The suspensions were freeze thawed three times and finally centrifuged at 10.000 r.p.m. for 10 minutes at 4°C. MPO activity in the resulting supernatant was assayed by mixing 25 *μ*L of 3,3′-5,5′-tetramethylbenzidine (TMB, Sigma Chemical Co., USA) prepared in dimethylsulfoxide (DMSO, Merck, USA), in a final concentration of 1.6 mM, with addition of 100 *μ*L H_2_O_2_ dissolved in phosphate buffer (pH 5.4) containing 0.5% HTAB, in a final concentration of 0.003% (v/v), and 25 *μ*L of the supernatant from the processed tissue sample. The assay was carried out in a 96-well microplate and was started by adding H_2_O_2_ to the supernatant sample and TMB solution, and incubated for 5 minutes at 37°C. The reaction was terminated by adding 100 *μ*L 4 M H_2_SO_4_ at 4° C and was quantified colorimetrically at 450 nm in a spectrophotometer (SPECTRAmax PLUS-Molecular Devices). MPO assay was also performed in samples collected from a naive group (nonligated, nontreated rats, *n* = 6). Results were expressed as change in optical density (OD) per gram (g) of wet tissue.

### 2.4. Assessment of Blood Leukocyte Number

On the 11th day of tooth ligation and under anesthesia, blood samples (30 *μ*L) were collected from femoral vein of control and drug treated-animals. Counting of whole blood cells (cells/mm^3^) used a mixture of 20 *μ*L blood samples + 380 *μ*L of Turk Blue solution in a Neubauer chamber under a light microscope (200-fold magnification). For specific cell counting, a smear of the animal blood (10 *μ*L) stained by Panotic L.B. (Haematological Dye System; 0.1% v/v, triarylmethane/0.1% v/v, xanthenes/0.1% v/v, thiazines, Laborclin) was used for analysis in a light microscope (1000-fold magnification).

### 2.5. Assessment of Local (Gingivomucosal) Cells

Specimens of gingivomucosal tissue from ligated (ipsilateral) and nonligated (contralateral) second molar teeth were removed as described in item 3 above (and) were fixed in 10% buffered formalin solution pH 7.2 during 48 hours. Samples were washed in tap water for 24 hours, dehydrated in serial alcohols, cleared, and embedded in paraffin. Paraffin blocks were cut in serial 4 *μ*m sections. Six images from different locations of the gingival tissue section were obtained at 400-fold magnification. An automatic macro recorder assembler (an algorithm of the KS300 software) was elaborated for capture, image processing, and segmentation, definition of morphometrical conditions and counts of all the nuclei contained in each image, as previously described [10–12]. Image processing techniques were applied in order to highlight the nucleus of the cells. Segmentation permitted the separation of these nuclei from the cell cytoplasm and from other structures in the section, such as blood vessels and extracellular space, enabling the creation of a binary image containing these two locations, nucleus and other spaces. The nuclei from resident cells in the gingivae as well as newly recruited leukocytes were then counted. The figures so obtained thus represented the total number of cells in gingivae under each specified condition. An observer who was unaware of the tissue sample captured the images and made the measurements. The result of the six fields counted was totaled and represented the total number of cells present in the tissue sample/animal.

### 2.6. Drug Administration Protocols

Either celecoxib (12 mg/kg/day; Searle & Co., Puerto Rico; *n* = 6 rats) or morphine (1 mg/kg/day; Merk, USA; *n* = 4 rats) was administered, in two different groups of rats, by subcutaneous (s.c.) bolus injection into a skin fold in the dorsal region (volume of 0.1 mL per 100 g body weight) from the 3rd to the 5th day of ligation. This protocol for drug treatment has been previously shown to be effective to relieve the inflammatory signs of periodontal disease under our conditions [11, 12]. A control group was administered with sterile saline, the vehicle of drugs used, by the same route and time of the drug-treated animals (*n* = 6 rats).

### 2.7. Statistical Analysis

The measurements are presented as mean ± Standard Error of the Mean (SEM) of the MPO activity (OD/g of wet tissue), number of blood cells (cells/mm^3^ and neutrophils/mm^3^), and number of total gingival cells (cells/fields) from 4–6 animals. Differences between means were evaluated by one-way ANOVA followed by the Student-Newman-Keuls test. Probabilities smaller than 5% (*P* < .05) were considered statistically significant.

## 3. Results

### 3.1. Macro- and Microscopic Features of Gingivomucosal Tissues From Periodontal-Diseased Animals

**Figure 1:**
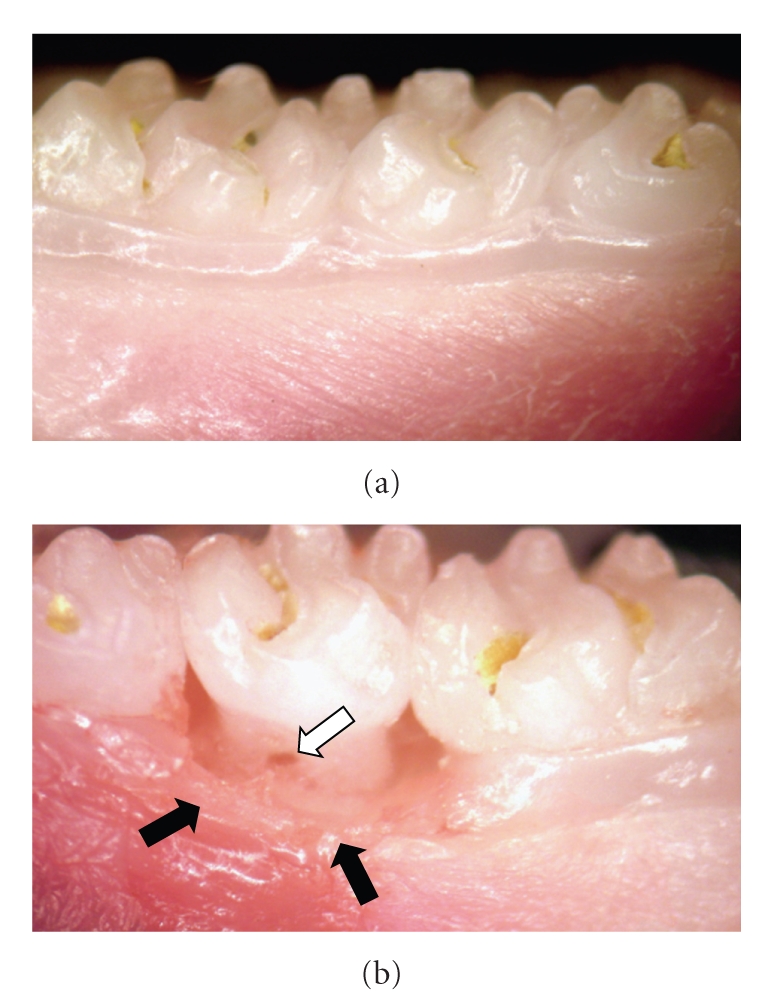
*Photographs from healthy (a) and periodontal-diseased teeth (b). *(a) Nonligated teeth do not show signs of inflammation. The periodontal supporting tissues present a healthy macroscopic aspect. Some left residues remained on the surface of the normal tooth. (b) Macroscopic signs of inflammation could be observed next to the second left molar of periodontal-diseased rats, after 11 days of ligation. Signs include redness, swelling, and destruction of the normal gingivomucosal tissue around the affected tooth (dark arrows), as well as a furcation lesion (white arrow). Original magnification: 15X.

**Figure 2:**
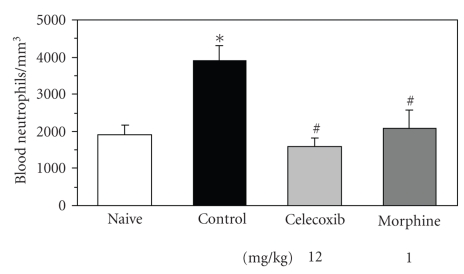
*Blood neutrophil number in whole blood from naïve, control, and treated periodontal-diseased rats.* For specific cell counting (%), a smear of the blood was prepared and stained by Panotic L.B. haematological dye system and observed under a light microscope (1000-fold). Celecoxib (12 mg/kg/day) or morphine (1 mg/kg/day) was subcutaneously administered for 3 consecutive days, from 3rd to 5th day after ligature placement as described in Materials and Methods. ∗ and # indicate significant difference (*P* < .05) in relation to naive and control group, respectively; one-way ANOVA followed by Student-Newman-Keuls test, 4–6 animals per group.

Panels (a) and (b) in Figure 1 show photographic recordings from a healthy (contra-lateral) and an affected (ipsilateral) tooth, respectively, from rats in which periodontal disease had been induced 11 days before. The placement of a silk ligature around the second rat maxillary molar tooth induced a progressive periodontal disease, as previously shown [11, 12]. In panel (b), of the same Figure, it can be observed that the supportive tissues around the inflicted tooth show clear signs of an ongoing-inflammatory process, including redness, increased volume, and destruction of the normal gingivomucosal structures as compared with the contralateral tooth from the same animals, shown in panel (a). Furcation lesion, a characteristic feature of periodontal destruction in this disease, was also detected, as indicated by the arrow in panel (b) (Figure 1). In contrast, macroscopic evaluation of gingivomucosal tissues from naive (not shown) or contralateral sites indicated a healthy tissue, with no signs of redness, attachment and/or bone loss (Panel (a), figure 1). In relation to microscopic changes, and although not reflected in total blood cell numbers obtained (Table 1), the number of circulating neutrophils in periodontal-diseased animals was twice (3,887 ± 469; Mean ± SEM) that found in naive animals (1,897 ± 298; Mean ± SEM; *P* < .05, ANOVA followed by Student-Newman-Keuls test), as shown in Figure 2.

### 3.2. Gingival Cell Number and Myeloperoxidase Activity in Tissues from Periodontal-Diseased Animals

**Figure 3:**
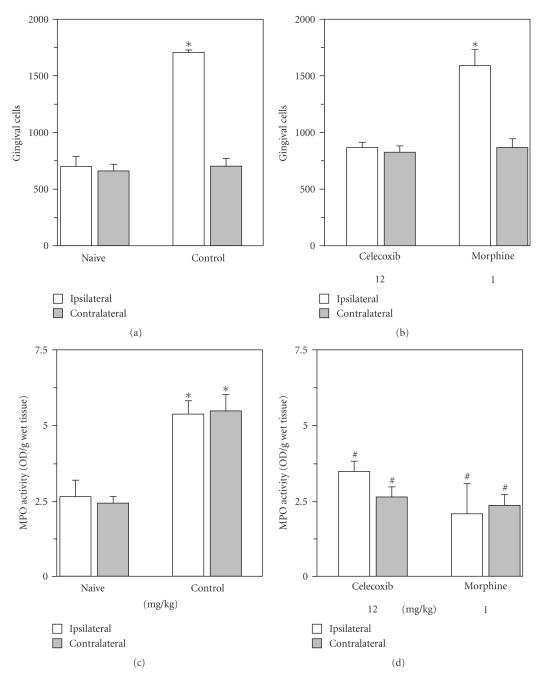
*Leukocyte recruitment and myeloperoxidase activity in gingivomucosal tissue surrounding rat molar tooth from naive, nontreated and periodontal-diseased treated rats.* Either celecoxib (12 mg/kg/day) or morphine (1 mg/kg/day) was administered subcutaneously (0.1 mL per 100 g body weight) once a day, for 3 consecutive days, from 3rd to 5th day after ligation. Animals were killed on the 11th day of ligation. Sterile physiological saline was given in the same volume and route of administration in control animals. Naive (N) animals were left nonligated. Gingivomucosal tissue samples of ligated (ipsilateral) and non-ligated (contralateral) second molar tooth (~20 mm^2^) were removed from naive, and drug-treated rats and were prepared for histological analysis (Panels (a) and (b)) and MPO activity (Panels (c) and (d)) as described in Materials and Methods. In panels (a) and (b) ∗ indicates a significant difference (*P* < .05) from contralateral side and in panels (c) and (d) ∗ and # indicate significant difference (*P* < .05) in relation to naive and control group, respectively; using one-way ANOVA followed by Student-Newman-Keuls test; 4–6 animals per group.

Morphometric analysis of gingival tissues surrounding ipsilateral second molar teeth from periodontal-challenged animals following 11 days of disease showed an approximate 2.5-fold increase in the total number of local cells (1,705 ± 21; Mean ± SEM; *P* < .05, ANOVA followed by Student-Newman-Keuls test) as compared with their contralateral counterpart (702 ± 66; Mean ± SEM) controls or naive (658 ± 60; Mean ± SEM) animals, as shown in panel (a) of Figure 3. Myeloperoxidase activity in gingival tissues obtained from diseased animals was shown to be also raised (5.35 ± 0.43; Mean ± SEM; *P* < .05, ANOVA followed by Student-Newman-Keuls test) in comparison with naive animals (2.62 ± 0.64; Mean ± SEM), by more than 2 fold, but surprisingly, in both ipsilateral and contralateral tissues (panel (c), Figure 3).

### 3.3. Effects of Celecoxib and Morphine in Periodontal-Diseased Animals

To assess their impact on MPO measurements, celecoxib and morphine, two drugs of already demonstrated efficacy on cell migration were investigated in the periodontal disease model. As shown in Figure 2, a remarkable specific decrease in circulating neutrophil numbers, which returned to naive levels, was observed in either celecoxib-(1575 ± 291; Mean ± SEM) or morphine-(2079 ± 581; Mean ± SEM) treated groups of animals (*P* < .05 in relation to controls, ANOVA followed by Student-Newman-Keuls test).

The morphometric analysis showed, in relation to control animals, that celecoxib treatment practically reduced to naive levels the (local) gingival number of cells, whereas morphine treatment did not (panel (b), Figure 3). Strikingly, both celecoxib and morphine treatments reduced to naive levels MPO content in ipsi and contralateral gingival tissues of periodontal-diseased animals (panel (d), Figure 3).

## 4. Discussion

Myeloperoxidase activity has been used as a good model to estimate neutrophil content in inflamed tissues [6, 15]; and for this reason, it has been widely used as a biomarker of inflammation. Moreover, myeloperoxidase activity was already reported to be increased in crevicular fluid of patients with infectious periodontal disease [7], and the concentration of this enzyme in periodontal tissues is correlated with the clinical state of periodontitis [16], which suggests that this measurement could be a good complement for diagnosis of such a condition. In fact, our results showed that, in rats compromised by the experimental periodontal disease, the myeloperoxidase activity was significantly higher at the sites of the chronic periodontitis, when compared to that of naive animals. These data are in accordance with many studies in literature, which have shown higher levels of myeloperoxidase in sites with periodontitis [16–18], total myeloperoxidase activity and myeloperoxidase concentration being present in significantly lower levels in healthy sites than in inflicted ones [19]. During periodontal disease development, a remarkable accumulation of neutrophils recruited from blood vessels can be observed in compromised periodontal tissues [20, 21]. Moreover, accumulation of neutrophils is related not only to host response to bacterial invasion but also to periodontal tissue destruction itself [20, 22]. Indeed, in the present study a raise (app. 2-fold increase) in blood neutrophils was observed in diseased animals along with a raise in local MPO content, but intriguingly, in both inflamed and noninflamed tissues from the diseased animals. It is our working hypothesis that all the specimens used for MPO determinations, from both the inflicted and noninflicted tooth neighboring gingival tissues, contained inflamed blood vessels full of circulating neutrophils, which accounted for a MPO positive response shown by periodontal-diseased animals. The intriguing issue was that histological evaluation of the gingivomucosal tissues surrounding rat teeth with evident signs of periodontal disease was also accompanied by a huge local content of cells, a finding not observed in tissues surrounding the contralateral teeth, which was in accordance with previous publications of our group [11, 12]. Therefore, local high myeloperoxidase content presented here did not correlate either with histological findings or with the objective macroscopic evaluation of nonchallenged tissues, clearly suggesting that the MPO model alone is not a good marker of local inflammation in an established chronic inflammation. Rather, it is a good marker of a systemic inflammation in this chronic inflammatory condition.

Likewise, it could be expected that systemic treatment using drugs of established mechanisms of action, such as celecoxib and morphine, a selective nonsteroidal anti-inflammatory drug [23], and a classical opioid agonist, respectively, would also reverse the detected increase in MPO activity. Indeed, our data confirmed and expanded previous works from our and other laboratories [12, 24], showing that celecoxib significantly reduced the number of blood neutrophils, which paralleled the reduction of cells present in inflamed gingival tissues [12], thus confirming in this model its activity as an anti-inflammatory drug. Moreover, the decrease in MPO content along with a reduced number in tissue cells by celecoxib suggests a more selective anti-inflammatory effect in this model than that shown by morphine, as discussed below.

In fact, it was intriguing to observe a reduction in the MPO levels also in rats treated with morphine, since it was previously shown that the opioid agonist was unable to affect the increased cell number in ligated sites, although it decreased other indicators of periodontal disease like bone and attachment loss, as shown here and in [11]. As for celecoxib, morphine demonstrated to have a qualitative effect on blood leukocytes since it decreased the number of neutrophils rather than affecting other leukocyte species. Since neutrophils were the predominant leukocyte in circulating cells, and neutrophils are considered to be key cells in periodontal destruction [22], the beneficial effect of morphine seen in compromised periodontal tissues was attributed to its property to reduce blood (circulating) neutrophils present in tissues rather than affecting the newly emigrated local cells. Our data are in line with other studies, which showed that morphine reduced both the MPO activity and the number of neutrophils, using an immunohistochemistry technique [25]. They also suggest that local neutrophils derived from a chronic inflammation are functionally distinct from circulating neutrophils, as the local cell numbers were not modified by systemic morphine treatment. A possible explanation for such findings would be, for instance, that the local activated neutrophils loose their opioid sensitive receptors, or alternatively, change the nature of their expressed opioid receptors. Notwithstanding, these possibilities were not further addressed in the present study. Taken together, our data clearly demonstrated that (1) blood neutrophil number, (2) local total cell number, and (3) MPO activity allow the detection of a differential profile of beneficial effects seen between celecoxib and morphine in this model of chronic inflammation. Therefore, we propose that this triad would be suitable to detect useful newer anti-inflammatory drugs in periodontal disease. 

In conclusion, our findings confirm the association between a high content of MPO and the presence of inflammation in tissues. Notwithstanding, they also indicate that, being done in a chronic condition, where the extravascular content cannot be strictly defined, MPO content reflects more the existence of a systemic inflammation, rather than a local inflammatory condition.

## Figures and Tables

**Table 1:** Leukocyte number in blood collected from untreated control and treated periodontal-diseased rats.

Group	Blood leukocytes (total cells/mm^3^; Mean ± SEM)
Naive	9,462 ± 352
Control	10,575 ± 1,213
Celecoxib	9,237 ± 936
Morphine	11,612 ± 226

On the 11th day of tooth ligation, naive, drug-treated (celecoxib at 12 mg/kg/day and morphine at 1 mg/kg/day), and saline treated groups of animals were anesthetized with 23 mg/kg xylazine and 116 mg/kg ketamine hydrochloride, and blood samples were obtained by femoral vein bleeding. Whole blood cell counting (cells/mm^3^) was performed using a mixture of 20 *μ*L of blood samples + 380 *μ*L of Turk Blue in a Neubauer chamber under a light microscope (x 200-fold). Celecoxib and morphine were systemically administered for 3 consecutive days, from 3rd to 5th day after ligature placement as described in Materials and Methods. Statistical analysis: *P* > .05, one-way ANOVA followed by Student-Newman-Keuls test, 4–6 animals per group.
